# Cell Metabolism and DNA Repair Pathways: Implications for Cancer Therapy

**DOI:** 10.3389/fcell.2021.633305

**Published:** 2021-03-23

**Authors:** Thais Sobanski, Maddison Rose, Amila Suraweera, Kenneth O’Byrne, Derek J. Richard, Emma Bolderson

**Affiliations:** ^1^Cancer and Ageing Research Program, Centre for Genomics and Personalised Health, Translational Research Institute (TRI), Queensland University of Technology (QUT), Brisbane, QLD, Australia; ^2^Princess Alexandra Hospital, Brisbane, QLD, Australia

**Keywords:** warburg effect, tumor metabolic reprogramming, homologous recombination, non-homologous end-joining, DNA repair, glycolysis, cell metabolism

## Abstract

DNA repair and metabolic pathways are vital to maintain cellular homeostasis in normal human cells. Both of these pathways, however, undergo extensive changes during tumorigenesis, including modifications that promote rapid growth, genetic heterogeneity, and survival. While these two areas of research have remained relatively distinct, there is growing evidence that the pathways are interdependent and intrinsically linked. Therapeutic interventions that target metabolism or DNA repair systems have entered clinical practice in recent years, highlighting the potential of targeting these pathways in cancer. Further exploration of the links between metabolic and DNA repair pathways may open new therapeutic avenues in the future. Here, we discuss the dependence of DNA repair processes upon cellular metabolism; including the production of nucleotides required for repair, the necessity of metabolic pathways for the chromatin remodeling required for DNA repair, and the ways in which metabolism itself can induce and prevent DNA damage. We will also discuss the roles of metabolic proteins in DNA repair and, conversely, how DNA repair proteins can impact upon cell metabolism. Finally, we will discuss how further research may open therapeutic avenues in the treatment of cancer.

## Introduction

DNA repair and metabolic pathways are vital to maintain cellular homeostasis. Under normal cellular conditions, DNA repair proteins can maintain genomic stability following exposure to exogenous and endogenous genotoxic insults. When growing in normal physiological conditions, cells predominately rely on the TCA cycle to generate ATP and other essential precursors for cellular processes. However, it has been well established that tumor cells are more likely to generate energy via glycolysis and hyperactivate their DNA damage response pathways, both of which promote the uncontrolled proliferative, survival and cellular growth pathways (Warburg, 1925). It was initially proposed that these two mechanisms operate independently within the cell; however, recent studies suggest a link between DNA repair and glycolysis. For instance, several independent studies have suggested novel roles for glycolytic proteins in DNA repair pathways, largely based on the observation that several glycolytic proteins, including Hexokinase II, Fumarase and ATP-citrate lyase (ACLY), migrate to the nucleus following exposure to genomic stress (van Vugt, 2017; Ohba et al., 2020; Hitosugi et al., 2012; Yuan et al., 2010). Several studies have also suggested glycolysis may be involved in maintaining genome stability, given that the glycolytic pathway provides metabolites which play an essential role in DNA metabolism. For example, the pentose phosphate pathway (PPP) utilizes the glycolysis intermediate, glucose-6-phosphate, to ultimately enable the biosynthesis of nucleotides via the generation of ribose-5-phosphate. Despite this, the interaction between DNA repair pathways and glycolysis remains unclear. Metabolic products from glycolysis, such as L- and D-lactate also play a role in DNA repair by decreasing chromatin compaction and subsequently increasing transcription of key genes involved in DNA DSB (double-strand break) repair (Wagner et al., 2015). Here, we will review the peer-reviewed evidence linking metabolism and DNA repair and how these processes may lead to radio- and chemo-resistance in tumor cells.

### The Warburg Effect and Tumor Metabolic Reprogramming

High glucose intake is a characteristic shared amongst most solid tumors, and this phenomenon was first described in 1920 by Otto Warburg (Warburg et al., 1927). This observation, referred to as the Warburg effect, describes how cancer cells shift their predominate metabolic pathway from oxidative phosphorylation to anaerobic glycolysis, consequently producing high levels of lactic acid via fermentation (Warburg et al., 1927; Warburg, 1956). Recently, studies have demonstrated that elevated lactic acid production may induce resistance to major anti-cancer therapies, including radiation and chemotherapy, via numerous mechanisms. Furthermore, the upregulated production of lactic acid contributes to the development of an acidic tumor microenvironment, which has been associated with increased metastatic capacity and growth rate in a subset of aggressive tumors (Turkcan et al., 2019).

In the early studies of Warburg’s effect, it was thought that cancer cells experience mitochondrial dysfunction via the “irreversible injuring of respiration,” as cancer cells downregulate oxidative phosphorylation during the tricarboxylic acid cycle (TCA, also known as the Krebs cycle) (Warburg, 1956). However, subsequent investigations of mitochondrial functionality in tumor cells revealed that the majority of tumor cells possess functional mitochondria, and can still undergo oxidative phosphorylation (Zong et al., 2016). This led to speculation as to why cancer cells with functional mitochondria preferentially convert excess pyruvate to lactate, instead of utilizing oxidative phosphorylation to more efficiently produce ATP.

As altered metabolic features are observed commonly across many cancer subtypes, reprogrammed metabolism is considered one of Pavlova and Thompson’s hallmarks of cancer (Pavlova and Thompson, 2016). For example, increased glucose uptake has been observed in a variety of tumor contexts and has been shown to negatively correlate with tumor prognostic markers and be involved in chemo- and radio-resistance mechanisms. This has been clinically exploited using ^18^F-deoxyglucose-positron emission tomography (FDG-PET) based imaging, where a radioactive fluorine-labeled glucose analog it utilized to diagnose and stage tumor progression (Spermon et al., 2002).

### DNA Repair Pathways and Their Relationship to Tumor Therapies

In tumor cells that undergo metabolic reprogramming, there is an observable increase in the activation of the DNA damage response pathways, which subsequently trigger nucleotide synthesis and anabolic glucose metabolism (Tong et al., 2009). DNA damage response pathways are highly active in tumor cells, subsequently promoting their rapid growth and survival. The DNA damage response consists of several DNA repair pathways, and each pathway represents a specific mechanism to repair a specific type of DNA damage. The initiation and progression of repair pathways is considered a spatiotemporally regulated process in which proteins move toward DNA damage sites, following the remodeling of the chromatin (van Attikum and Gasser, 2009; Gospodinov and Herceg, 2013). DNA damage may be induced by several endogenous sources such as DNA double-strand breaks and oxidative stress induced by reactive oxygen species, resulting from cellular metabolism. DNA damage may also result from exogenous sources, for example nucleotide damage from UV light or oxidative damage and DNA strand breaks caused by ionizing radiation (Jackson and Bartek, 2009; Tubbs and Nussenzweig, 2017). In order to maintain the integrity of genome, in human cells there are several types of DNA repair processes, classified into five major pathways including base excision repair (BER), nucleotide excision repair (NER), mismatch repair (MMR), non-homologous end-joining (NHEJ), and homology-directed repair (HDR) (Kalluri, 2016; Roos et al., 2016; Chatterjee and Walker, 2017). In addition to having a critical role in maintenance of genome integrity, alterations in the expression, and function of DNA repair proteins are a major mediator of tumor responses to chemo- and radiotherapy, which commonly function by inducing DNA damage in tumor cells. Here, we will briefly discuss the relevance of each repair pathway on tumor sensitivity to chemo- and radiotherapies, but further detail can be found in the following review (Minchom et al., 2018).

In terms of chemo- and radiotherapy, DSB repair via NHEJ and HDR is an important consideration, since many therapies, including radiotherapy, topoisomerase inhibitors, such as doxorubicin, and PARP inhibitors, induce DNA DSBs. Therefore, the defective functioning of DSB repair pathways can significantly influence the tumor response to these therapies. For example mutations or decreased expression of the Breast Cancer Associated 1 and 2 (BRCA1 and BRCA2) proteins can lead to defects in the HDR of DNA DSBs, sensitizing tumor cells to PARP inhibitors and radiotherapy that induce lesions that require HDR for repair (Rose et al., 2020). Conversely, upregulation of DNA DSB repair proteins in the NHEJ pathway can also induce resistance to these DSB-inducing therapies, due to the tumor cells ability to rapidly repair DNA damage and therefore avoid induction of cell death (Jensen and Rothenberg, 2020). BER removes and repairs damaged bases within the DNA. The capacity of cells to perform BER is also of relevance to tumor therapy as the anti-tumor agents temozolomide, pemetrexed, or floxuridine induce DNA lesions of N7mG, uracil, or 5-FU, respectively, all of which can be recognized and repaired by the BER pathway (Storr et al., 2011). Upregulation or down regulation of the BER pathway can lead to resistance or sensitivity, respectively, to these agents. Several inhibitors of the BER pathway are also in development (Grundy et al., 2020).

In the process of MMR, proteins recognize mismatched bases in DNA which arise from processes such as replication. MMR proteins identify, excise and replace these mismatched bases with the correct pairing base. Mutations in the MMR genes Mlh1 and Msh2 are associated with the human colon cancer-prone syndrome, Lynch Syndrome [also known as hereditary non-polyposis colorectal cancer (HNPCC)], but MMR genes are also frequently mutated in other cancers. Tumors with mutated Mlh1 and Msh2 in colon tumors were historically targeted with methotrexate, which leads to the accumulation of oxidative damage. However, due to the high number of somatic mutations found in MMR-deficient tumors, which can contribute to stimulation of the immune system, immunotherapy is showing potential to become the preferred therapy for tumors with defects in MMR (Le et al., 2015).

The nucleotide excision repair pathway recognizes damaged nucleotides including pyrimidine dimers, intrastrand crosslinks, and bulky adducts. Alkylating agents, such as platinum compounds like cisplatin are commonly used to treat many types of cancers and induce intrastrand crosslinks within the DNA, activating the NER pathway. Expression of the NER proteins, including ERCC1 are correlated with sensitivity to platinum agents in multiple tumor types due to an inability to resolve DNA crosslinks (Arora et al., 2010).

Therefore, although alterations in DNA repair pathways contribute to the development of tumors, and can lead to resistance to tumor therapies, they also hold huge potential as the next generation targets for the treatment of many cancer types. Due to the metabolic reprogramming in tumor cells, it is likely that targeting cellular metabolism may also be advantageous. The current literature supporting a link between metabolic reprogramming and the DNA damage response pathways will be further explored below.

### The Requirement of the Pentose Phosphate Pathway (PPP) and G6PD Protein in DNA Damage Prevention and DNA Repair Processes

The pentose phosphate pathway is a parallel pathway to glycolysis and generates pentoses and NADPH, together with ribose-5-phosphate, a precursor for nucleotide synthesis (Patra and Hay, 2014). The PPP is upregulated in several tumor types and regulates various functions that promote tumor growth, including DNA metabolism and cell proliferation (Mori-Iwamoto et al., 2007; Chan et al., 2013; Catanzaro et al., 2015). In non-carcinogenic cells, the PPP pathway is responsible for generating the bulk of nucleotides through salvage pathways, which recycle existing nucleosides and nucleobases. Although, a portion of nucleotide synthesis also takes place via *de novo* synthesis pathways to produce purine and pyrimidine rings to sustain rapid DNA metabolism (Mori-Iwamoto et al., 2007). Supporting this, highly proliferative cells, such as tumor cells, are more likely to use *de novo* nucleotide synthesis pathways over the salvage pathways to maintain the increased production of nucleotides and other macromolecules. The *de novo* nucleotide synthesis pathways maintain nucleic acid and protein synthesis, along with other cellular activities, to meet the high metabolic requirements of cancer cells (Kilstrup et al., 2005; Villa et al., 2019).

**FIGURE 1:**
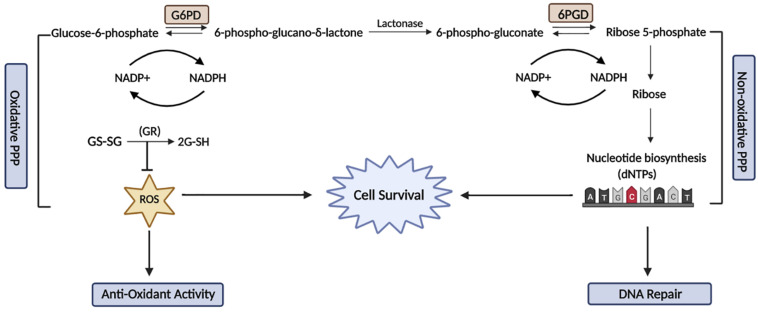
The relationship between the Pentose Phosphate Pathway and DNA damage and repair. The PPP comprises of two phases known as the oxidative and non-oxidative phases. The oxidative phase is responsible for the conversion of glucose-6-phosphate to ribose-5-phosphate, which releases NADPH to maintain the cellular redox balance and also reduces oxidative damage. In the non-oxidative phase, the activity of a key enzyme, G6PD is stimulated by ATM to promote the production of NADPH and nucleotide synthesis. The activation of G6PD is essential to maintain a reduced cellular environment and also synthesises nucleotide precursors for DNA damage repair. This figure was created with BioRender.com.

The PPP pathway consists of an oxidative and a non-oxidative phase: the oxidative phase generates NADPH that is used for reductive biosynthetic reactions, such as fatty acid synthesis and the prevention of oxidative stress by detoxification of oxygen species (ROS). The non-oxidative arms of the PPP produce ribose-5-phosphatase, which is then further metabolized for the production of nucleotides (Figure 1). The PPP pathway occurs in parallel to glycolysis, diverging from glycolysis at glucose-6-phosphate (G6P), which is involved in the oxidation of glucose to provide the building blocks for anabolic pathways (D’Urso et al., 1983). Alternatively, under conditions of high reductive demand cancer cells have the capacity to divert glucose-6-phosphate dehydrogenase (G6PD) into the PPP pathway to maintain the constant generation of NADPH and nucleotides (Bokun et al., 1987). Downregulation of NADPH production renders tumor cells more susceptible to oxidative DNA damage, as NADPH functions as a major cofactor for glutathione (GS) and cytochrome p450 reductase, which is essential for maintaining the cellular redox balance.

Cells lacking G6PD are more sensitive to oxidative damage and therefore have increased sensitivity to ionizing radiation (IR), which in addition to inducing DNA strand breaks, also causes oxidative damage (Tuttle et al., 2000). G6PD is essential in sustaining a balanced pool of nucleotides in response to DNA damage and promotes PPP-mediated nucleotide synthesis. Furthermore, a study showed that Ataxia Telangiectasia Mutated (ATM), a key DNA damage protein, activates the PPP pathway through G6PD to promote antioxidant defense mechanisms and DNA repair activity via nucleotide production under stressed conditions (Cosentino et al., 2011). This suggests that G6PD activity is likely to also be required for the repair of DNA damage and maintaining DNA integrity (Zhang et al., 2016).

The wild type tumor suppressor protein, p53, has also been shown to downregulate the PPP via directly reducing G6PD activity (Jiang et al., 2011). However, inhibition of ATM is also known to downregulate p53 expression, subsequently promoting the constitutive upregulation of the PPP via G6PD upregulation too, consequently restoring dNTP levels in cancer cells and facilitating cellular proliferation (Aird et al., 2015). G6PD is the rate-limiting enzyme that regulates oxidative PPP and therefore controls the flux of dNTP production required for DNA replication and maintaining genome stability. As such, G6PD is also required to suppress dNTP-enhanced mutagenesis. Overall, the altered cellular metabolic flux induced by G6PD during metabolic reprogramming enables the more rapid repair of DNA lesions, promoting resistance to conventional therapies such as radiation, and cellular growth advantages (Leick and Levis, 2017).

Activating mutations of FMS-like tyrosine kinase 3 (FLT3) have been shown to drive the initiation and progression of acute myeloid leukemia (AML). As such, inhibition of FLT3 was suggested to be a promising treatment for AML; however, targeting FLT3 as a monotherapy did not achieve long term remission (Leick and Levis, 2017). In contrast, a genome-wide RNA interference (RNAi)-based screen found that inhibition of the ATM/G6PD pathway in combination with FLT3 inhibition was synthetically lethal (Gregory et al., 2016). Thus, the simultaneous targeting of ATM-mediated G6PD regulation and inhibition of up-regulated nucleotide synthesis following chemotherapy induced stress may offer a new treatment option by decreasing DNA repair capacity. Following this, the targeting of key enzymes that regulate PPP also potentiated the effect of conventional therapies to selectively suppress cancer cell growth. For example, treatment with the glycolysis inhibitors 2-Deoxy-D-glucose (2DG) and 6-aminonicotinamide (6AN) has been shown to increase radio-sensitivity in squamous carcinoma cell lines (Khaitan et al., 2006; Sharma et al., 2012). In addition, this suggests that the inhibition of PPP or G6PD in combination with DNA damage inducing chemotherapies, such as 5-fluorouracil (5-FU) and doxorubicin, may restore chemosensitivity in cancer cells.

### ATM and DNA-PK Kinases Play a Key Role in Cellular Energy Sensing

**FIGURE 2:**
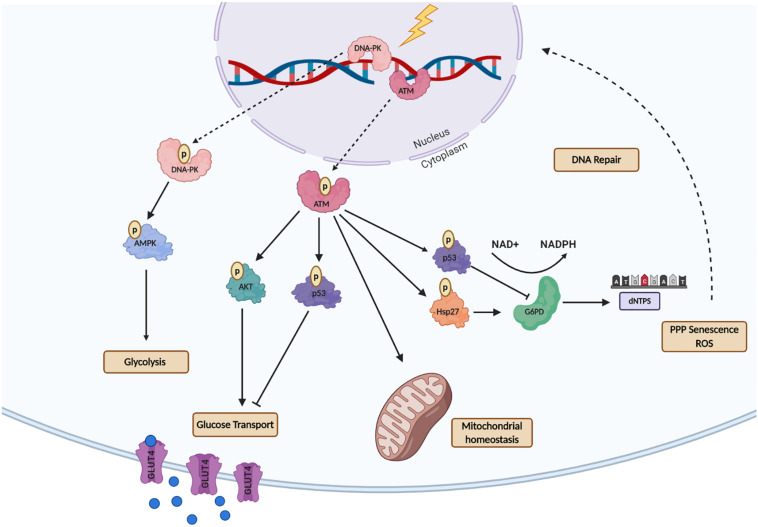
A schematic representation of the Ataxia-Telangiectasia Mutated- (ATM) and DNA-Dependent Kinase- (DNA-PK) mediated regulation of metabolic processes after DNA Damage. ATM activates multiple downstream proteins regulating cell cycle arrest, DNA repair and cellular metabolism. ATM activates the tumor suppressor p53 which decreases GLUT recruitment, glycolysis, and dNTP production. ATM non-canonical function is essential for repairing mitochondrial genome defects to maintain mitochondrial homeostasis. For maintenance of energy production ATM activates AKT to promote glucose recruitment to the nucleus via GLUT4-mediated transport. ATM also activates G6PD though Hsp27, as an alternative mechanism to produce nucleotides for DNA metabolism. DNA-PK following energy depletion or metabolic rewiring promotes glycolysis through AMP signaling. This figure was created with BioRender.com.

Ataxia telangiectasia mutated and DNA-dependent kinases (DNA-PK) are key proteins that recognize DNA damage and initiate DNA damage repair signaling (Mirzayans et al., 2006; Marechal and Zou, 2013). Upon activation by DNA-damage, these kinases generate a cascade of phosphorylation events that regulate the recruitment and activity of many downstream effector proteins to repair DNA double-strand breaks (DSBs) (Cosentino et al., 2011; Aird et al., 2015). ATM is generally considered to form a homodimer, while the active DNA-PK complex is comprised of the DNA-PK catalytic subunit bound to the Ku70/80 heterodimer. Several studies have shown that both DNA-PK and ATM are also involved in cellular metabolism rewiring after DNA damage for energy supply by activating of glucose transporter (GLUT4) thought AKT, maintenance of mitochondrial homeostasis and increased nucleotide production for DNA metabolism (Figure 2). This is particularly evident in individuals with Ataxia-Telangiectasia syndrome (A-T), which results from mutations in the ATM gene. These individuals exhibit alterations in cellular metabolism, including the dysfunction of enzymes involved in glucose metabolism and mitochondrial function (Sharma et al., 2014; Volkow et al., 2014).

Besides its primary function in the recognition of DNA damage, ATM functions as a metabolic stress sensor, identifying reductions in the energy levels of tumor cells, subsequently promoting increased PPP activity, which can lead to increased cancer cell survival and resistance to conventional therapies (Krüger and Ralser, 2011). Additionally, there is a growing quantity of evidence showing that ATM also regulates the translocation of glucose transporter 4 (GLUT4), which in part explains why patients with A-T syndrome tend to present high incidences of type 2 diabetes mellitus (Halaby et al., 2008). It is known that cytoplasmic ATM is an insulin-responsive protein that activates AKT following insulin treatment, and inhibition of ATM leads to downregulation of AKT activity that in turn downregulates the GLUT4 glucose transporter protein (Halaby et al., 2008). A recent study found that loss of ATM-mediated p53 Ser18 (murine Ser15) phosphorylation led to increased metabolic stress and insulin resistance (Armata et al., 2010). Additionally, ATM was also shown to enhance glycolysis in breast cancer cells via GLUT1-phosphorylation and PKM2 up-regulation, increasing lactate production. High levels of lactate were found to promote tumor invasion through lactate-mediated metabolic coupling (Sun et al., 2019). These recent studies suggest that ATM is essential for glycolysis homeostasis as it regulates key metabolic proteins that are responsible for the maintenance of glucose levels such as glucose transporters.

DNA-dependent kinases is best known for recognizing DSBs and initiating DNA repair responses by activation of the NHEJ pathway. DNA-PK is an abundant, cytoplasmatic protein that migrates to the nucleus after DNA damage (Yang et al., 2014). There is also growing evidence indicating that DNA-PK may function to regulate metabolic homeostasis (Weterings and Chen, 2008; Lieber, 2010). Similar to ATM, DNA-PK also functions as a metabolic stress sensor and regulates AMPK (AMP-activated protein kinase) in response to energy depletion or metabolic stress in mammalian cells. AMPK is an essential protein that recognizes when energy production is low. It has been shown that inhibition of the DNA-PK catalytic subunit, decreases AMPK activity in response to energy deprivation.

Cell starvation leads to the phosphorylation of AMPKα (Thr172) and acetyl-CoA carboxylase (ACC). However, the inhibition of DNA-PKcs inhibits AMPK phosphorylation, thereby disrupting the sensing of glucose metabolism by AMPK. In addition, it was shown that DNA-PKcs directly interacts with the energy monitoring regulatory subunit of AMPK (Amatya et al., 2012). This finding suggests that DNA-PK is essential for activating AMPK under low energy levels as a result of glucose deprivation in mammalian cells. Similarly, another study confirmed that DNA-PKcs is a positive regulator of AMPK activity and was found to phosphorylate two residues on AMPKγ (S192 and T284) (Puustinen et al., 2020). Conversely, another study showed that an aging-related increase in DNA-PKcs activity led to decreased AMPK activity, via phosphorylation-mediated inhibition of Hsp90 chaperone activity toward AMPKαα2 (Park et al., 2017). It is also possible that the interaction between DNA-PKcs and AMPK may depend on cellular context as DNA-PKcs itself is regulated by the cellular metabolic state and may decline as individuals’ age.

Autophagy is the process by which damaged proteins or organelles are degraded by the lysosome, this provides a mechanism to recycle cellular components providing macromolecular precursors and energy for cellular metabolism. Autophagy is generally classified into five defined steps: initiation, vesicle nucleation, vesicle elongation, vesicle fusion and cargo degradation. The regulation of autophagy by metabolic proteins and vice versa have been well charactered but there is also mounting evidence that DNA repair is also regulated by autophagy [reviewed in Hewitt and Korolchuk (2017)]. Some studies suggest that DNA repair is inhibited by autophagy, but other studies propose that autophagy promotes DNA repair (Bae and Guan, 2011; Liu et al., 2015). In order to explain this discrepancy, it has been hypothesized that following low levels of DNA damage, autophagy may promote DNA repair, while severe DNA damage may lead to autophagy-dependent degradation of DNA repair proteins to promote apoptosis (Guo and Ying, 2020). Autophagy has been shown to be initiated by AMPK activation and/or inhibition of the metabolic sensor Mammalian Target of Rapamycin Complex 1 (mTORC1), establishing another link between metabolism and DNA repair pathways (Kim et al., 2011). As discussed above the DNA-PK-dependent regulation of AMPK may also provide a feedback loop to regulate autophagy in the context of DNA repair (Puustinen et al., 2020).

### Key Metabolic Enzymes That Play a Role in DNA Repair and Resistance to Chemo- and Radiotherapies (Summarized in Table 1)

### Phosphoglycerate Mutase Enzyme (PGAM)

The Phosphoglycerate mutase 1 (PGAM1) is a key glycolytic enzyme that coordinates different metabolic process including glycolysis, PPP, and serine biosynthesis in cancer cells. As a result of its dynamic role in metabolic coordination, PGAM1 is overexpressed in several cancer types, including gliomas, oral carcinomas and pancreatic cancers (Liu et al., 2008, 2018; Zhang et al., 2017). For example, PGAM1 activity directly regulates the PPP and the resulting production of nucleotides, promoting cancer cell proliferation and tumor resistance to conventional therapies. Indirectly, PGAM1 contributes to DNA repair activity in cancer cells by the upregulation of glycolysis and/or nucleotide synthesis (Ohba et al., 2020). However, it was also found that PGAM1 plays a direct role in DNA repair as its activity was required for the repair of DNA double-strand breaks via homologous recombination (HR). Its role in HR was shown to be through regulating the stability of CTBP-interacting protein (CtIP), which is essential for the recruitment of Rad51 to sites of damage to facilitate filament formation (Qu et al., 2017). Complementary studies in gliomas cells demonstrated that depletion of PGAM1 also led to defective DNA damage signaling, including ATM autophosphorylation and phosphorylation of its downstream substrates. This led to disrupted DSB repair and subsequent sensitivity to IR, suggesting that PGAM1 may be a potential therapeutic target in gliomas (Ohba et al., 2020).

**TABLE 1:** The effects of metabolic proteins and metabolites on DNA repair.

Metabolic protein	Canonical function	Non-canonical function	Inhibitor	Clinical stage	Observations	DNA repair pathways	References
PGAM phosphoglycerate mutase enzyme	Catalyzes the conversion of 3-phosphoglycerate (3PG) to (2PG)	Maintains the stability of CTBP-interacting protein (CtIP) Activates DDR pathway via the regulation of WIP1 activity	PGM1-0004A	Preclinical data	Reduces tumor growth *in vitro*/vivo	HR	Hatzivassiliou et al., 2005; Wellen et al., 2009; Keller et al., 2014; Koerner et al., 2017
PKM2 pyruvate kinase M2	Pyruvate production	Binds to the promoter of HIF increasing its transcriptional activity Promotes DSB repair as ATM phosphorylates PKM2;	TLN-232 (Thallion)	Preclinical data	Decreases aerobic glycolysis in some tumor models	HR	Hatzivassiliou et al., 2005; Wellen et al., 2009; Keller et al., 2014; Koerner et al., 2017
FH fumarate hydratase/fumarase	Catalyzes the conversion of fumarate to malate	Produces fumarate inhibiting the demethylase KDM2B increasing H3 methylation Phosphorylates DNA-PK	Miconazole nitrate (MN)	Preclinical data	Reduces DNA repair activity Enhances the cytotoxicity of cisplatin (CDDP)	NHEJ	Hatzivassiliou et al., 2005; Wellen et al., 2009; Keller et al., 2014; Koerner et al., 2017
ACLY ATP-citrate lyase	Production of acetyl-CoA	Production of ACLY increases histone acetylation, promoting DNA repair Increases the expression of genes promoting cell cycle progression and cell proliferation	SB-204990 (Furan carboxylate derivatives)	Preclinical data	Anti-proliferative activity in several cancer cell lines Tumor growth inhibition	HR	Seltzer et al., 2010; Wang et al., 2010; Tardito et al., 2015; Fu et al., 2019
GS glutamine synthetase	Catalyzes the conversion of glutamate and ammonia into glutamine	Enhances DNA repair via *de novo* nucleotide synthesis	Glutaminase inhibitor (CB839)	Clinical data	Sensitizes malignant cells expressing mutant IDH1 to GLS1-targeting agents Studies in Leukemia and solid tumors	HR	Seltzer et al., 2010; Wang et al., 2010; Tardito et al., 2015; Fu et al., 2019
PFKB3 6-phosphofructo-2-kinase/fructose-2,6-Biphosphatase 3	PFKB catalyzes the synthesis of F6P to F2,6BP	Nuclear PFKB3 drives cancer cell proliferation Promotes recruitment of HR factors	KAN0438757	Preclinical data	Induces radiosensitivity in transformed cells	HR	Yalcin et al., 2009; Gustafsson et al., 2018
L/D-lactate	Cellular energy source	Promotes histone hyperacetylation and decreased chromatin compactness leading to enhanced DNA repair activity Induces the expression of genes involved in DNA repair, such as upregulation of DNA-PK	1-(phenylseleno)-4 (trifluoromethyl) benzene (PSTMB) Galloflavin	Preclinical data	Inhibits the cellular growth of several tumor cell lines; Blocks aerobic glycolysis and induces cell death by triggering apoptosis	HR and NHEJ	Hunt et al., 2007; Manerba et al., 2012; Sonveaux et al., 2012; Kim et al., 2019

### Fumarase/Fumarate Hydratase (FH)

Under normal cellular conditions FH localizes mainly in the cellular cytosol and mitochondria (Kornberg and Krebs, 1957). A study in yeast demonstrated that following the induction of DNA damage, FH moves to the nucleus and functions as a DNA repair protein to promote the repair of DSBs (Yogev et al., 2010). In human cells, FH plays a similar role in DNA repair and was found to be a substrate of DNA-PK, which phosphorylates FH at Threonine 236. This stimulates the local generation of fumarate near DSBs, which inhibits the activity of the histone demethylase, KDM2B (Jiang et al., 2015). Subsequently, increasing the level of Histone H3 lysine 36 dimethylation which has been shown to facilitate the recruitment of DNA-PK to DSB sites and subsequently facilitate NHEJ activity (Fnu et al., 2011). A recently study showed that depletion of fumarase prolonged the interaction of Mre11 at sites of DSBs, delaying the progression of the HR pathway (Leshets et al., 2018). In addition, increased FH expression also disrupts HR by the inhibition of two key lysine demethylases (KDM4A and KDM4B) in Leiomyomatosis Renal Cell Cancer (HLRCC). This syndrome is classified as a familial DNA repair deficiency syndrome, as these patients carry a germline mutation in FH leading to defective responses to DNA damage and results in a higher predisposition for cancer development (Sulkowski et al., 2018).

### Pyruvate Kinase M2 (PKM2)

Pyruvate kinase is an enzyme that converts phosphoenolpyruvate and ADP into pyruvate to generate ATP, and its activity is essential for the maintenance of glucose homeostasis. Pyruvate kinase M2 (PKM2) is highly expressed in cancer cells and a master regulator of tumor metabolic reprogramming (Wu et al., 2016; Zheng et al., 2018). Under normal conditions PKM2 is an abundant cytosolic protein that upon certain cellular stress, such as ultraviolet light (UV) or H_2_O_2_, migrates to the nucleus (Stetak et al., 2007). The migration of PKM2 to the nucleus has been associated with its non-metabolic functions, as PKM2 was found to phosphorylate several nuclear proteins, including histone H3 (Yang et al., 2014). It was also reported that nuclear PKM2 interacts with histone H2AX after DNA damage, and that PKM2 could directly phosphorylate H2AX on serine 139, one of the first phosphorylation events following DNA damage. Furthermore, replacement of wild type PKM2 with a kinase dead form led to increased chromosomal aberrations following DNA damage. Collectively, this reveals PKM2 as a novel modulator for genomic instability in tumor cells (Xia et al., 2017). As part of its non-metabolic activity it was also recently uncovered that PKM2 directly promotes DSB repair, as ATM phosphorylates PKM2 at Threonine 328 (T328) to induce the nuclear accumulation of PKM2 (Matsuoka et al., 2007). This ATM-mediated phosphorylation of PKM2 was shown to be required for efficient homologous recombination (HR) through the recruitment of CtIP at the site of DSBs. Additionally, the disruption of the ATM-PKM2-CtIP axis interaction was shown to sensitize tumor cells to a variety of DNA-damaging agents, including PARP inhibitors (Sizemore et al., 2018).

### ATP-Citrate Lyase (ACLY)

ATP-citrate lyase is a nuclear-cytoplasmic enzyme that utilizes acetyl-CoA to generate citrate, and plays a crucial role in conserving the global histone acetylation in mammalian cells (Wellen et al., 2009). ACLY deficiency has been shown to result in defective DSB repair, due to the depletion of acetyl-CoA pools and reduction in acetylated histones at sites of DSBs (Kumari et al., 2019). Supporting this Sivanand et al. showed that nuclear acetyl-CoA played a role in HR and following DNA damage ACLY was phosphorylated at Serine 455, in an ATM- and AKT-dependent manner. Additionally, ACLY phosphorylation and nuclear localization were necessary to promote BRCA1 recruitment in order for HR to occur (Sivanand et al., 2017). Thus, acetyl-CoA production by ACLY is critical for the repair of DNA DSBs.

### Glutamine Synthetase (GS)

Glutamine synthetase (GS) is an enzyme that catalyzes the conversion of glutamate and ammonia into glutamine. Transcriptome analyses revealed that GS is responsible for the metabolic reprogramming that occurs in tumor cells, as GS activity was shown to enhance DNA repair via *de novo* nucleotide synthesis (Kalluri, 2016). Further analyses revealed that knockdown of GS delayed DNA repair due to impaired nucleotide metabolism, which led to increased radio-sensitivity. HR was impaired in GS depleted cells further supporting a role for GS in DSB repair. Collectively, these findings suggest glutamine synthase plays a similar role to G6PD in DNA repair, as its upregulation increases nucleotide synthesis leading increased DSB repair capacity.

### The Role of Metabolic Reprogramming in Tumor Cell Chemo- and Radio-Resistance

Radiotherapy remains a key anti-cancer therapy, with over 50% of patients undergoing radiation treatment as a monotherapy or in combination with other therapies (Kalluri, 2016). However, a significant proportion of patients experience resistance to conventional radiotherapy. Studies have demonstrated that the likelihood of radio-resistance is influenced by several factors, including metabolic changes and the upregulation of DNA repair pathways (Dwarkanath et al., 2001; Schwarz et al., 2008). Metabolic reprogramming may enable tumor cells to enhance nucleotide synthesis through the upregulation of the PPP, subsequently promoting resistance to traditional anti-cancer therapies (Zhao et al., 2016; Yin et al., 2017). Supporting this, several studies have shown that upregulation of metabolic enzymes or metabolic processes increases the activity of DNA repair pathways. For example, as a result of elevated glycolytic activity, some tumors generate a high level of lactate, which can promote cisplatin-resistance through increased DNA repair activity (Wagner et al., 2015). As previously discussed, several metabolic enzymes from glycolysis and PPP play a direct role in DNA repair pathways, and inhibition of key enzymes of both pathways not only inhibited cellular proliferation but also restored radio-sensitivity by decreasing DNA repair activity. The link between radio-resistance and altered metabolism is not fully understood but several studies suggest that decreasing the metabolic activity of the key enzymes involved in the PPP and glycolysis pathways could restore the sensitivity of resistant tumors to conventional therapies.

In ovarian cancer, three glycolytic enzymes, HK2, PFK, and PKM2, have been suggested to be promising targets due to their positive correlation with chemo- and radio-resistance via anti-apoptotic and cell survival mechanisms (Li et al., 2015; Zhang et al., 2018; Lin et al., 2019). There are four isoforms of PK; however, the PKM2 isoform is a key regulator of glycolysis in cancer cells and is thus the most prominent potential candidate for restoring sensitivity to therapies. Supporting this, the inhibition of PKM2 in cervical cancer cells leads to decreased cell viability, G2/M cell cycle arrest, and promotes apoptosis (Lin et al., 2019). Furthermore, inhibition of PKM2 may induce radio-sensitivity, as demonstrated by a study which found that PKM2 depletion decreases AKT and PDK1 phosphorylation to subsequently promote radio-sensitivity in NSCLCs (Yuan et al., 2016). Similar to LDHA, miR-133 overexpression inhibits the expression of PKM2, which restores the sensitivity of radio-resistant lung cancer cells, offering a potential new treatment option for these radio-resistant tumors (Liu et al., 2016).

Hexokinase 2 (HK2) is a key glycolytic enzyme that catalyzes the first essential step of glucose metabolism. Like many other glycolytic proteins, HK2 is highly expressed in several tumor types (Anderson et al., 2017; Wu et al., 2017). Similar to other metabolic proteins, inhibition of HK2 has been shown to increase radio-sensitivity in cancer cells (Vartanian et al., 2016). 2-deoxy-D-glucose (2-DG) is an inhibitor of glucose metabolism, that is phosphorylated by Hexokinase to produce 2-deoxyglucose-6-phosphate. The intracellular accumulation of this metabolite inhibits hexokinase activity and therefore ATP production via glycolysis. Significantly, the anti-proliferative effects of 2-DG have been demonstrated in numerous preclinical studies (Giammarioli et al., 2012; Zhang et al., 2015). 2-DG has also been shown to be an effective sensitizer in several tumor types, including gliomas and lung carcinomas (Dwarkanath et al., 2001; Singh et al., 2019). Additionally, combining 2-DG with chemotherapy has already shown promising results in its ability to restore the sensitivity of chemo-resistance cells. A recent study analyzed the effect of combination treatment with 2-DG and carboplatin chemotherapy in high stage and recurrent ovarian clear cell carcinoma (OCC), and found that 2-DG in combination with carboplatin and cisplatin chemotherapy increased efficacy in chemo-resistant ovarian tumor cell lines and patient-derived xenograft models (Zhang et al., 2015; Khan et al., 2020). Thus, the combination of 2-DG with both radio- and chemotherapy drugs improves tumor cell sensitivity; however, the underlying mechanism for the restoration of sensitivity to therapy remains largely unknown.

The glucose transporter GLUT1 is involved with the early steps of glucose uptake and metabolism. GLUT1 is overexpressed in many types of cancers and has been evaluated as a potential target for anti-cancer drugs (Wincewicz et al., 2010; Koch et al., 2015; Kim and Chang, 2019). Depletion of GLUT1 using small interfering RNA (siRNA) was shown to increase the radiosensitivity of laryngeal cancer cells and led to the downregulation of DNA repair. Similarly, restoration of radio-sensitivity was observed when antisense oligonucleotides (AS-ODNs) were used to inhibit GLUT1 activity in laryngeal carcinoma cells (Chan et al., 2004; Yan et al., 2013). In breast cancer, a synthetic inhibitor of GLUT1 known as WZB117, was demonstrated to radio-sensitize cancer by increasing the level of intracellular ROS, thereby inhibiting tumor growth (Zhao et al., 2016). Thus, inhibition of GLUT1 has therapeutic potential as an intervention to overcome cellular radio-resistance.

L-lactate is produced by glycolysis and is found to be expressed in high quantities in malignant tumors. High lactate levels have also been associated with resistance to clinical chemotherapeutics in numerous cancer subtypes. Recently, studies have shown that lactate can inhibit the activity of histone deacetylases (HDACs), which leads to changes in chromatin structure and transcription (Wagner et al., 2015, 2017). HDACs remove acetyl groups from histones, and their inhibition results in increased acetylation of histones, which are generally associated with a more open chromatin structure to promote transcription. This open chromatin state has also been suggested to increase accessibility of DNA repair proteins to sites of damage, in turn increasing the rates of DNA repair (Tamburini and Tyler, 2005). A study showed that lactate also modulates chromatin compaction in cervical cancer, leading to the up-regulation of DNA-PKcs (Wagner et al., 2015). Thus, the characteristic increase in lactate levels in tumor cells results in increased DNA repair activity, which has been shown to enhance radio-resistance in cervical carcinoma. Additionally, L/D-lactate was shown to increase the rate of γ-H2AX foci resolution after irradiation and induce cisplatin resistance, consistent with the up-regulation of DNA repair pathways (Wagner et al., 2015). Lactate dehydrogenase (LDHA) is a key metabolic protein found in almost all human tissues that is required for the conversion of pyruvate to lactic acid, playing an important role in the final steps of glycolysis. Increased expression of LDHA induces hypoxic environments that are associated with tumor metastases, poor overall survival, and radio-resistance in several tumor types, including prostate and bladder cancers (Koukourakis et al., 2009, 2014, 2016). Based on these findings, it can be suggested that the inhibition of LDHA activity may confer sensitivity in tumor cells to DNA damaging agents (Manerba et al., 2015). Supporting this, a soluble adenylate cyclase (sAC) that promotes the release of LDHA, led to the activation of the BRAF/ERK1/2 signaling pathway and consequently increased radio-resistance in prostate cancer cells (Flacke et al., 2013; Appukuttan et al., 2014). Treatment of prostate cancer cells with an LHDA-specific inhibitor, FX-11, reduced the activity of DNA repair proteins, improving cellular sensitivity to radiotherapy (Hao et al., 2016). Another study demonstrated that miR-34a overexpression inhibits LDHA and restored radio-sensitivity in hepatocellular carcinoma cells (Li et al., 2016). Based on these findings, it has been suggested that targeting LDHA via miR-34a may provide a mechanism to restore sensitivity to therapies in radio-resistant tumors (Li et al., 2016). Lactate influx and efflux is mediated by four members of the solute carrier 16a family Monocarboxylate transporters (MCT1-4). These proteins control the transport of lactate across the plasma membrane, effectively controlling lactate homeostasis. Given that high lactate levels confer chemo- and radioresistance, MCTs may also represent an effective mechanism to target lactate levels in tumor cells and increase sensitivity to DNA damaging agents (Halestrap, 2012).

## Conclusion

Genomic instability and metabolic reprogramming are central components in cancer development and evolution. These changes in chromatin structure, DNA repair enzyme expression and mutation allow the cancer cells to develop genetic heterogeneity, which in turn can promote evolution and metastasis. The metabolic changes allow cancer cells to increase growth rates, adapt to the rapidly changing external environment and to reduce the reliance on oxygen. It has become increasingly apparent that these two processes do not exist in isolation, but instead are mutually dependent. This raises the notion that the cell must adapt more globally to small changes in metabolism. While the DNA damage signaling kinases regulate the metabolic state of the cell, the opposite is also true. This generates a regulatory loop that ensures that changes in one pathway have compensatory changes in the other pathways. It must be considered that this link, and the peer reviewed studies supporting the link, have predominantly occurred in cancer cell lines and studies. It is likely these studies sit at the extreme of changes that have occurred in genomic instability and metabolism, as these cells have adapted to a metabolic and genetic state that gives them a growth and survival advantage. Understanding how these processes function under normal physiological cell conditions and indeed how they may drive the process of aging and other age-related diseases needs to be further addressed. During the aging process, the DNA repair capacity of cells declines and also undergo metabolic changes induced by cellular and endocrine changes. Understanding, how these changes in metabolism and DNA repair capacity under the normal process of aging may shed further light on why cancers form in the first place. In cancer, further studies may also identify new therapeutic targets that can target both metabolism and DNA repair concurrently.

## Author Contributions

TS made the figures. All authors listed have made a substantial, direct and intellectual contribution to the work, and approved it for publication.

## Conflict of Interest

KO and DR are founders of CARP Pharmaceuticals. EB, DR, and KO are founders of Carpe Vitae Pharmaceuticals. EB, KO, and DR are inventors on patent applications filed by Queensland University of Technology. The remaining authors declare that the research was conducted in the absence of any commercial or financial relationships that could be construed as a potential conflict of interest.

## Abbreviations

ACLY: ATP-citrate lyase
ATM: ataxia telangiectasia mutated
DDR: DNA damage response
DNA-PK: DNA-dependent kinases
2DG: 2-Deoxy-D-glucose
FDG-PET: ^18^F-deoxyglucose-positron emission tomography
FH: fumarate hydratase/fumarase
FLT3: FMS-like tyrosine kinase 3
GLUT4: glucose transporter 4
GS: glutamine synthetase
G6P: glucose-6-phosphate
G6PD: glucose-6-phosphate dehydrogenase
HK2: hexokinase 2
PFKB3: 6-phosphofructo-2-kinase/fructose-2,6-biphosphatase 3
PGAM: phosphoglycerate mutase enzyme
PKM2: pyruvate kinase M2
TCA: tricarboxylic acid cycle.
